# Exploring the relationship between built environment and multidimensional street market vitality: Insights from urban villages in Shenzhen using multi-source data

**DOI:** 10.1371/journal.pone.0332905

**Published:** 2025-09-19

**Authors:** Yueyi Tan, Jusheng Song, Yunxi Bai

**Affiliations:** School of Architecture, Harbin Institute of Technology Shenzhen, Shenzhen, China; Shenyang Jianzhu University, CHINA

## Abstract

Street markets play a vital role in revitalizing cities, especially within urban villages, which are unique neighborhoods characterized by dense migrant populations and complex social dynamics. This study addresses the underexplored relationship between street market vitality and the built environment, aiming to promote sustainable urban growth and inclusivity. A comprehensive framework embraces both macro-structural and micro-experiential perspectives, integrating urban morphology, functionality, and human perception indicators, to investigate the relationship between multidimensional vitality and the built environment in Shangxia Sha urban village street markets, Shenzhen, China. Utilizing multi-source data, geographic information systems, street view semantic segmentation, and space syntax, the study applies Geographically and Temporally Weighted Regression (GTWR) and Multiscale Geographically Weighted Regression (MGWR) models to explore spatiotemporal and multi-scale spatial heterogeneity. Findings reveal the pivotal role of urban functionality in shaping multidimensional vitality. Specifically, social vitality is most positively correlated with bus convenience and most negatively with street length. In terms of commercial vitality, point of interest (POI) density shows a strong positive relationship, while life service facility density is most negatively associated. This research provides insights into how the living street scenes shaped by low-income and migrant populations interact with the urban environment, offering guidance for fostering urban inclusivity and diverse communities.

## 1. Introduction

Street markets in rapidly urbanizing Chinese cities like Shenzhen are critical components of vibrant living environments, particularly in urban villages where they serve as hubs of commercial and social activity [1,2]. According to data released by the China Development Institute in September 2023, as of the end of 2022, Shenzhen contained 2,042 urban village units, which collectively accommodated approximately 60% of the city’s total resident population [3]. The street markets within these urban villages play a critical role in meeting the daily needs of this population by providing accessible and affordable necessities and services. Furthermore, these markets, which integrate commercial activities with community interactions in public spaces, are essential in meeting the diverse needs of working-class and lower-income residents [4,5]. However, as economic and demographic shifts occur, these markets face increasing challenges in adapting to the evolving demands of local residents. Specifically, they are confronted with challenges such as spatial compression caused by rapid urban redevelopment, conflicts between informal economic practices and formal urban management, as well as pressures from the rise of e-commerce and changing consumption patterns, all of which require traditional street markets to adapt in order to meet the evolving demands of local residents. In a new wave of urban renewal, promoting street market vitality has become an increasingly important goal. Understanding the dynamics of street market vitality in urban villages has been crucial for generating valuable insights into fostering inclusive and dynamic urban communities.

The street market is a multi-layered urban environment, encompassing streets, surrounding buildings, vendors, open stalls, and their social networks [6]. Due to this complexity, urban scholars commonly study street markets from an integrated perspective, viewing them as a fusion of urban public and commercial spaces [7,8]. However, systematic research on street market vitality remains limited. Streets, as an important component of street markets, have had their vitality as a key focus of research for a long time [9]. Since the 1960s, studies on street vitality have provided valuable insights into analyzing market vitality and its influencing factors. Recently, street vitality has been widely recognized as a phenomenon closely linked to human interactions within specific spatial-temporal contexts influenced by the built environment [10]. Its core lies in diverse human activities, representing social vitality, which can be quantified by population density and is affected by urban morphology, functions, and human perceptions [11]. These theories have formed a crucial foundation for uncovering the relationship between street market vitality and the built environment.

Existing studies on street vitality, though insightful, mostly focus on homogeneous urban streets and often overlook the dynamic and multidimensional nature of street market ecosystems. To fully understand market vitality, several underexplored issues need attention. Firstly, most studies emphasize a single dimension of vitality, particularly social vitality [9], despite street markets being complex spaces where commercial and social vitality intersect. Research specifically addressing this multidimensionality is limited. Secondly, traditional methods such as Ordinary Least Squares (OLS), spatial lag models, and Geographically Weighted Regression (GWR) [12,13] often fail to capture the multi-scale spatial non-stationarity and temporal dynamics of street market vitality. Thirdly, previous research has focused on macro-level built environment features, like functionality and morphology [14], neglecting the interaction between macro-planning and micro-informal practices in street markets. This lack of an integrated perspective is a significant gap. Lastly, traditional analyses rely on time-consuming field surveys [15], which are limited by sampling bias and external factors, making large-scale application difficult.

The advancement of urban data, such as location-based services (LBS) data, spatial big data, social media check-ins, user-generated content, open-source built environment data, and POI [10], along the mature application of emerging technologies like machine learning and geocomputation in urban research [9], provides granular and comprehensive insights into the multidimensional vibrancy of street markets and the built environment features that shape macro structures and micro experiences. At the same time, the emergence of GTWR model, which captures spatiotemporal heterogeneity, and MGWR model, which considers multiscale spatial heterogeneity, make it possible to explore the complex spatiotemporal relationship between multidimensional vibrancy and the built environment. Motivated by these advancements, this paper aims to systematically analyze how the built environment affects street market social and commercial vitality across different spatial and temporal dimensions, identifying key environmental factors that enhance vitality. The research will offer city planner insights to optimize multidimensional vitality, drive sustainable urban growth and improve quality of life.

The structure of this paper is as follows: Section 2 defines key concepts and reviews related studies. Section 3 details the research methodology, including design, study area, variables, data, and regression models. Section 4 examines the diverse impacts of built environment factors on social and commercial vitality, highlighting their spatiotemporal heterogeneity and multiscale effects. Section 5 discusses the research contributions and policy implications. The paper concludes by summarizing key findings, limitations, and future steps.

## 2. Literature review

### 2.1. Roles and impacts of street markets across contexts

Street markets can be defined as dynamic urban spaces comprising vendors, open-air stalls, surrounding infrastructure like adjacent buildings and streets, and social networks forming a holistic market ecosystem [6]. In China, these markets have evolved into central hubs for trade, leisure, and social interaction, with historical roots in early settlements. Street markets have been widely studied for their multifaceted roles in supporting city life. Scholars have explored these markets as both material and social spaces, emphasizing their unique contributions to the social, economic, and cultural dimensions of urban life.

As material spaces, street markets are integral to the urban fabric, reflecting the city’s architectural and cultural heritage. Jacobs [16] emphasizes that the vibrancy of urban streets depends on the dynamic interplay between people, space, and activities, with diverse space being central to their success. Ashihara [17] introduces the concept of “street aesthetics,” emphasizing that the visual and architectural qualities of streets shape their role in the urban environment. Gehl [18] highlights the importance of improving spaces like plazas and street corners to support meaningful public engagement.

As social spaces, street markets are seen as vital for public life and community interaction. Kohn [19] defines them as environments that blend public and private spheres, offering social, economic, and cultural value. Sharon Zukin’s comparative studies of global urban streetscapes illustrate the ecological and cultural significance of street markets as carriers of local identity and diversity [8]. Traditional markets draw newcomers and evolve into global hubs, fostering intercultural connections through a diverse array of products, traders, and customers [7]. A series of empirical studies demonstrate that street markets significantly contribute to economic support and social cohesion across different cultural contexts. Research on markets like Moore Street and Maxwell Street reveals their multifaceted contributions to livelihoods, urban planning, and socio-economic vitality, as well as their role in immigrant integration [6,20]. In London, street markets enhance the well-being of low-income families and minority groups by providing affordable goods and fostering social cohesion [21]. Similarly, studies in Hong Kong demonstrate that street markets are instrumental in forming supportive communities for vulnerable populations by facilitating social interaction [22].

Existing research predominantly focuses on street markets in urban centers or traditional communities, while exploration of street markets within the unique context of urban villages remains relatively limited. Urban villages, a phenomenon unique to China’s urbanization process, emerge when rural settlements located in or near urban centers are gradually surrounded by modern urban development [2]. These areas retain rural land ownership and management systems and are often characterized by high-density construction and underdeveloped infrastructure. Despite these challenges, urban villages provide affordable housing and feature vibrant street markets offering inexpensive goods, thus accommodating large numbers of migrant workers and low-income groups [23]. However, studies of public spaces in urban villages offer valuable insights. These informal public spaces, including street markets, vegetable markets, various shops, and street corners, facilitate migrant integration and enhance the intensity of social and economic activities [4]. The aesthetic appeal of urban villages partly stems from their vibrant lifestyles and informal landscapes, closely tied to these public spaces [24]. As such, street markets within urban villages reflect the cultural diversity and economic vitality unique to these areas, underscoring their importance in the broader context of China’s urbanization.

Although previous research has highlighted the significance of street markets in urban environments, it often remains at the qualitative analysis stage due to technological limitations, lacking in quantitative assessment of their multidimensional vitality. In recent years, advancements in urban data, such as LBS data and user-generated content, have enabled real-time analysis of population distribution, activity patterns, and consumer behavior. These technologies offer new opportunities to explore the social and commercial vitality of street markets in urban villages.

### 2.2. The street market vitality and its built environment

Researchers often study the complex nature of street markets within the context of commercial and urban public spaces, with a focus on streets to analyze vitality. This integration of research on streets, commercial spaces, and public spaces aids in understanding street market vitality and its influencing factors. Since the 1960s, urban studies have explored urban and street vitality from various perspectives. Jacobs [16] introduced the concept of urban vitality, emphasizing the role of human activities and their connection to living spaces, with street vitality as a key manifestation. Inspired by Jacobs, subsequent research adopted a human-centered perspective. Gehl [18] promoted pedestrian-friendly environments to enhance vitality, while Lynch [25] highlighted the importance of diversity, social connections, and openness. Street market vitality, specifically, refers to the economic activity and social interaction they facilitate [26]. As essential components of urban environments, street markets serve as spaces where commerce and community life intersect, contributing to the overall dynamism of city life. Socially, they are lively spaces where diverse populations interact, creating a sense of community and belonging [22]. These interactions are facilitated by the market’s physical and social infrastructure, including layout, accessibility, and communal spaces that encourage gathering and engagement. Economically, vibrant street markets are marked by diverse vendors offering goods and services, benefiting both businesses and consumers [6]. These studies collectively establish the link between human activity intensity and the built environment, offering key insights into their relationship.

To deeply understand street market vitality, exploring the impact of the built environment is crucial. Jacobs [16] first proposed that factors such as density, land use mix, accessibility, and block size influence urban vitality from the perspective of urban morphology and function. Since then, the influence of urban morphology on vitality has been a core issue in urban studies. Advances in urban form tools and geographic information systems have provided new methods for describing, classifying, and representing street morphological characteristics. The book “The Social Logic of Space” introduces “space syntax” theory, showing how spatial configuration influences social interactions, offering a theoretical framework to understand and enhance urban vitality [27]. Subsequent research has adopted a comprehensive approach to studying the influence of urban morphology on downtown vitality by integrating factors such as floor area ratio (FAR), density, and connectivity into a composite index [28]. Additionally, some studies have focused on the unique impacts of morphological factors in different cities. A study in downtown Shanghai indicated that building height and density affect social and economic vitality [29]. Comparative research between American and Asian cities has further revealed how different density and location characteristics impact vitality [30].

Urban functionality is a crucial factor influencing street vitality, as emphasized by renowned urban scholars like Jacobs and Montgomery. Various researchers examined the applicability of the functional indicators proposed by Jacobs across different cities. An empirical study in Seoul supported Jacobs’ views, finding that walking activities were related to the six conditions of urban vitality outlined by Jacobs [16]. A study in Chaozhou, China, confirmed that the diversity of street functions positively impacts vitality on weekdays [31]. Improving functional density and land use mix is considered a more effective strategy for enhancing street vitality in Shanghai and Osaka [12,29]. However, changes in urban environments and socio-economic conditions over time have made Jacobs’ theories less universally applicable. Some research has shown that land use mix does not significantly affect street vitality [32]. Researchers have also explored various indicators including functional mixture, POI density, convenience of street facilities, accessibility of transportation facilities, development intensity, and their correlation with street vitality [9,33–35].

Recent empirical research increasingly examines how human perception indicators affect street vitality. Utilizing deep learning algorithms for image processing, researchers can precisely analyze street view images, focusing on elements like greenery, sky rate, walkability, and street facilities. Findings suggest that greenery often negatively impacts street vitality [9,11], whereas the facility visibility has a positive effect [9]. Sky rate and walkability show varied results, with some studies indicating positive impacts [12] and others showing inconsistent outcomes [31]. This approach emphasizes understanding street vitality through individual perception and experience, serving as a micro-level complement to macro-level studies of urban morphology and functionality.

While the influence of the built environment on street and urban vitality is widely acknowledged, studies have not reached a consensus on their specific relationship. Research varies in terms of correlation and association strength. For instance, building density and FAR impact vitality differently across scales and development stages [36]. Some studies find a positive link between transit station density and urban vitality [12], while others do not. These differences reflect variations across cities and studies, influenced by spatial, social, and cultural contexts. Thus, research should consider local conditions to analyze key environmental factors and thresholds.

Quantitative studies on street vitality and the built environment have evolved from small-scale manual research to large-scale urban studies using multi-source big data. Early studies relied on methods like field observation, behavioral analysis, and surveys [37,38], offering deep insights but facing limitations such as high costs, subjectivity, and small data scales. The emergence of open-source built environment data, POI data, and street view images has made large-scale analyses of the built environment possible, encompassing urban morphology, functionality, and human perception indicators. Recent advancements in big data, including spatial economic data, mobile signaling, social media check-ins, LBS data, and street view images, have enabled more comprehensive and dynamic analyses of the multidimensional vitality of streets. These data sources measure various aspects of street vitality from different angles, particularly focusing on social vitality, and cater to diverse research needs with real-time updates and broader coverage.

For social vitality, spatial economic data, such as light data, effectively illustrates nighttime vitality by revealing the intensity and spatial distribution of human activities. In assessing neighborhood and street vitality, mobile signaling, social media check-ins, and LBS data are invaluable. For instance, user check-in frequencies provide insights into community engagement [39,40], while LBS data helps analyze population dynamics across various times and spaces, shedding light on street activity levels [9,11]. Street view data further enriches urban vitality studies. One study developed a comprehensive urban vitality index by employing deep learning techniques on street view images to count pedestrians and vehicles, integrating these findings with social media check-ins [12]. Another approach, the DLM-SVC model, infers street vitality through pedestrian counts and activity classifications derived from street view imagery [13]. For commercial vitality, small dining businesses serve as strong indicators of daytime economic activity [14]. Additionally, platforms like Dianping are used to map the spatial distribution of urban commercial vitality [41], offering a nuanced view of economic landscapes.

Despite advances in street vitality research, challenges remain in comprehensively studying street market vitality. Most studies emphasize social vitality, with few addressing economic aspects. Current indicators, such as the number of stores or the number of reviews on platforms like Dianping or Meituan, cannot fully reflect the commercial vitality of street markets and need improvement for more accurate evaluation. Streets are multi-layered environments where the bottom-up construction process is vital. Research often overlooks the human perception of street space, lacking integration with urban morphology and function. Additionally, the widely used global regression models fall short in capturing the complexity of street multidimensional vitality, necessitating the exploration of more refined models.

## 3. Methodology

### 3.1. Research design

**Fig 1:**
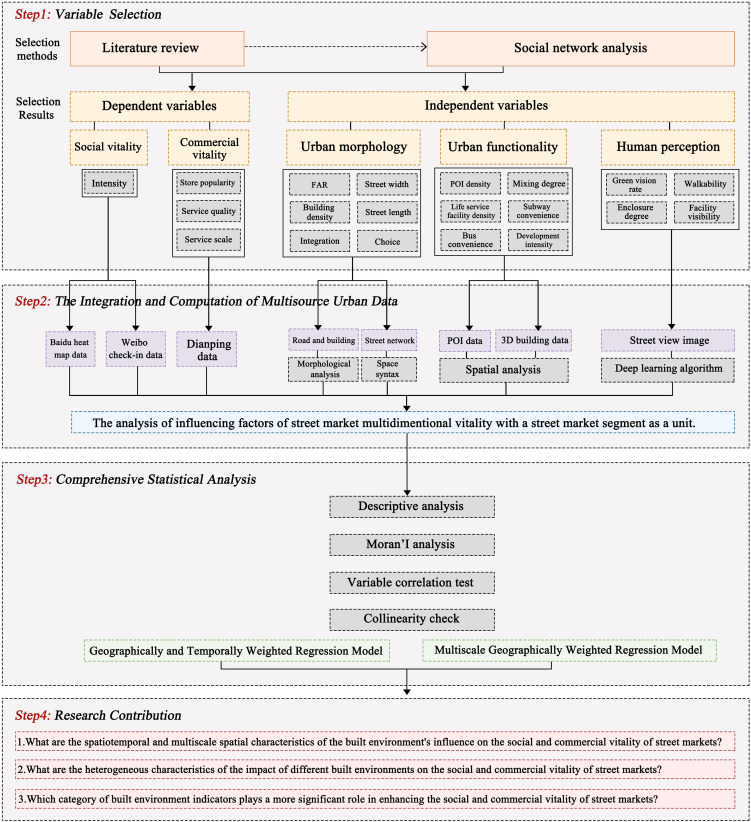
Research framework.

The research framework consists of four main sections (Fig 1). 1) Step 1: variable selection. This stage involves identifying research categories and indicators through a combination of literature review, field research, and social network analysis (SNA). Social vitality is measured through street market activity, while commercial vitality is evaluated through a composite indicator encompassing store popularity, service scale, and service quality. Independent variables include urban morphology, functionality, and human perception. 2) Step 2: integration and computation of multi-source urban data. This step integrates diverse datasets, including Baidu Huiyan population heat map data, Weibo check-in data, and Dianping data for vitality, alongside POI data, 3D building data, road network data, and street view images for the built environment. These data sources are analyzed using spatial analysis, syntactic analysis, and deep learning techniques. 3) Step 3: comprehensive statistical analysis. This stage begins with a descriptive analysis of the variables to provide foundational insights. Subsequently, GTWR models are employed to capture the spatial-temporal dynamics of social vitality, while MGWR models are used to explore the multiscale spatial impacts. 4) Step 4: research contributions. This section highlights the three primary contributions of the study, summarizing its key findings and implications.

### 3.2. Study area

**Fig 2:**
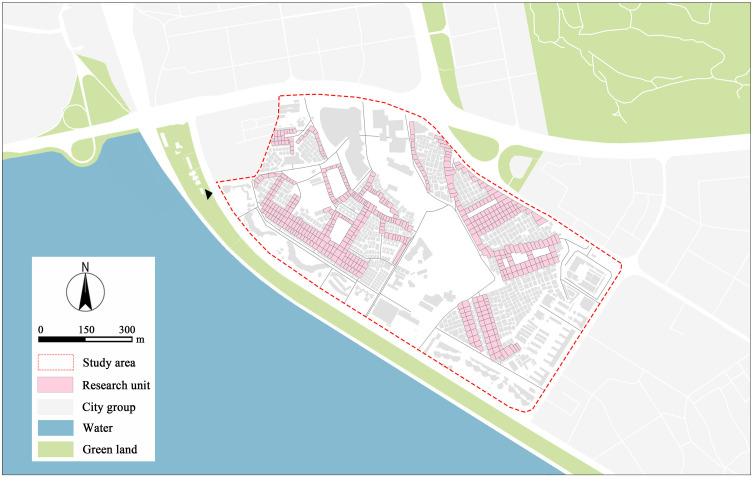
The study area and the spatial distribution of research units. The basemap, including roads and building outlines, is based on data from OpenStreetMap contributors (Open Database License, ODbL). Building outlines were further supplemented and corrected by the authors through field surveys. Colored research units, city group, water and green land were independently delineated and visualized by the authors in GIS according to the research objectives.

This study investigates the vibrancy and dynamics of street markets within the Shangxia Sha urban village in Shenzhen. Against the backdrop of Shenzhen’s rapid development, the traditional spatial forms and socio-cultural structures of Shangxia Sha have undergone significant transformations. Centered around its marketplaces, this urban village has developed a diverse social structure predominantly comprised of migrant populations, serving as a prototypical example of the interaction and evolution of urban village spaces and marketplaces in Shenzhen. Currently, Shangxia Sha covers approximately 71 hectares, accommodates around 100,000 residents, and is located in the city center, making it the largest urban village in Futian District (Fig 2). It retains the quintessential characteristics of urban villages, such as high-density housing, narrow streets, and vibrant markets. Its diverse population structure, which includes numerous migrant workers, low-income residents, and some original inhabitants, makes it an ideal case for exploring the impact of varied socioeconomic and cultural backgrounds on marketplace vitality. Shangxia Sha not only epitomizes urban village characteristics closely connected to urban cores but also showcases the high-density and highly functional mixed-use urban forms of the Greater Bay Area. This targeted research approach not only aids in understanding the dynamics of urban villages in Shenzhen and the Greater Bay Area but also provides a reference framework for urban studies in other regions.

No specific permits were required for fieldwork in Shangxia Sha urban village, as the area is publicly accessible and our research involved only non-invasive observations and analysis of publicly available data. No private or restricted areas were entered, and no human or animal subjects were involved.

### 3.3. Variables and data

#### 3.3.1. Research unit.

**Fig 3:**
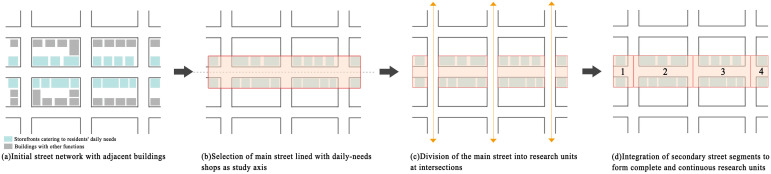
Delineation process of research units based on streets and adjacent storefronts.

In this study, streets and their adjacent storefronts are defined as fundamental research units. As shown in Fig 3, the delineation process for these units is as follows: First, a primary street densely lined with shops catering to residents’ daily needs is selected as the study axis. This street is divided into multiple independent research units based on its intersections with secondary streets. Each research unit consists of a segment of the main street interrupted by a secondary street and the storefronts on both sides of this segment. Second, to ensure continuity and spatial integrity of research units, we split the intersecting secondary streets into equal parts, to fully consider their impact on street vitality and the commercial environment. The divided portions of the secondary street are then respectively integrated into the preceding and succeeding research units, ensuring that each unit is spatially independent and functionally consistent. Ultimately, 508 research units were delineated within the Shangxia Sha urban village, primarily distributed along the main or central streets of the area, as shown in Fig 2.

#### 3.3.2. Dependent variable.

This study separately assesses social and commercial vitality. Social vitality represents the core and tangible aspect of street market vitality, while commercial vitality provides the foundation and prerequisite for its overall emergence.

Currently, LBS data like mobile phones and public transportation cards are widely used to measure social vitality [42], with GPS precision up to 10 meters, providing precise insights into spatial behavior [43]. This study utilizes Baidu Huiyan population heat map data and Weibo check-in data to comprehensively assess vitality. Baidu Huiyan population heat map data offer significant advantages due to their rapid updates and extensive geographic coverage. This Data provides real-time insights into urban population density and mobility, capturing dynamic crowd distribution and street activity intensity. As of December 2021, Baidu Maps handled over 130 billion location requests daily, covering more than 11 million kilometers of roads. Weibo check-in data complements Baidu Huiyan population heat map data by reflecting social interactions and engagement at specific locations. With 583 million monthly active users, Weibo effectively captures diverse urban social activities. By integrating these two datasets, we can conduct large-scale, multi-period, and detailed observations of street market activities, revealing spatial distribution and social behavior in urban areas, thereby enabling a comprehensive analysis of social vitality.

The data for this study were collected between March 11 and March 24, 2024. This period falls in early spring in Shenzhen, a season characterized by abundant outdoor and street activities. Importantly, the selected two-week window does not include any public holidays or major local festivals, thereby minimizing the potential influence of holiday-related fluctuations in pedestrian flow. This design helps to ensure that the measured vitality indicators capture typical urban dynamics and are not unduly influenced by atypical fluctuations in pedestrian activity. The data collection and processing methods are as follows: First, vitality data at 15-minute intervals from Baidu Maps Huiyan Urban Population Geospatial Big Data Platform and Weibo Open Platform during two weeks in March 2024 were collected. Second, hourly aggregation of 15-minute interval records was conducted to determine the combined hourly count per research unit, with each hour’s activity averaged over the same hour across 14 days. Third, data cleaning, filtering, and error modeling techniques were employed to enhance accuracy. Finally, the calculation method for street activity intensity is as follows:


$$Intensity=\frac{N_{pop}}{S_{unit}}$$ (1)

where ${\text{N}}_{pop}$ refers to the combined hourly count for each research unit, calculated as the sum of the number of unique devices invoking the Baidu Maps Location SDK and the number of Weibo check-ins, and $S_{unit}$ is the area of each research unit.

**Fig 4:**
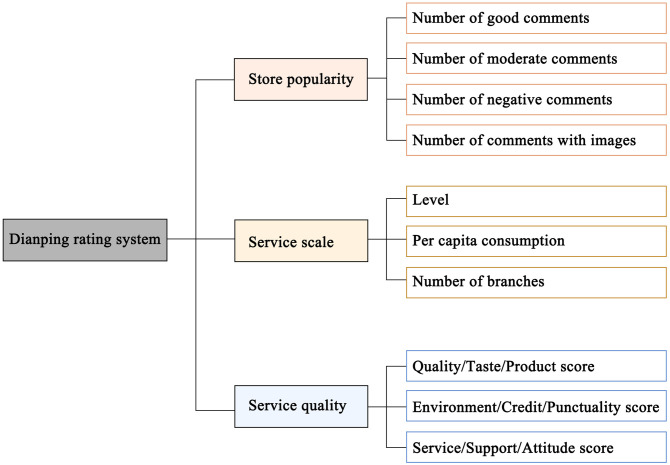
Dianping rating dimensions and corresponding sub-indicators.


To measure the commercial vitality of street markets in urban villages, which are marked by small-scale businesses like restaurants and retail shops, this study uses data from the Dianping app, renowned for its wide coverage and multidimensional and detailed user reviews of such enterprises [41]. Considering previous studies often use single indicators like store count and ratings [10], and given the multidimensional data provided by Dianping, we have selected store popularity, service quality, and service scale as key indicators to more effectively reflect commercial vitality. The specific secondary indicators are shown in Fig 4. By analyzing Dianping data from March 2024, the study identified 1,977 stores in Shangxia Sha urban village. After data cleaning, 794 facilities offering offline services, including food shopping, leisure and entertainment, and life services, were evaluated for their commercial vitality using the selected indicators. To ensure data suitability for factor analysis, the Kaiser-Meyer-Olkin (KMO) test and Bartlett’s test of sphericity were performed, yielding a KMO value of 0.785 and a p-value of 0.000, confirming appropriateness for principal component analysis. The initial factor loading matrix was rotated using variance maximization, with the eigenvalues of the three principal components exceeding 1, resulting in a cumulative variance contribution rate of 85.183%. These components effectively captured user ratings across different store types. In a relevant urban study [44], PCA-derived weights were used to construct composite indices based on the variance contribution of each principal component, and this approach was applied specifically in the assessment of commercial vitality. Based on this research, the composite score of user ratings was calculated using the model:

$$\text{V}={\text{W}}_{\text{1}}{\text{V}}_{\text{1}}+{\text{W}}_{\text{2}}{\text{V}}_{\text{2}}+{\text{W}}_{\text{3}}{\text{V}}_{\text{3}}$$ (2)

where ${\text{W}}_{\text{i}}$ is the coefficient of each principal component (variance contribution ratio), reflecting the relative importance of each dimension in explaining the overall variance in the data. ${\text{V}}_{\text{1}}$, ${\text{V}}_{\text{2}}$, ${\text{V}}_{\text{3}}$, are used to represent the store popularity, service scale and service quality, respectively. The purpose of involving indicator weights derived from principal component analysis is to provide an objective and data-driven method for quantifying the relative importance of each indicator dimension in the overall commercial vitality assessment. Unlike subjective weighting schemes, PCA weights are determined based on the actual variance each component explains in the multidimensional data, ensuring that indicators contributing more to the variability of user ratings have a proportionally greater impact on the composite score. This approach improves the robustness and scientific validity of the composite index by minimizing human bias and emphasizing the most informative aspects of the data. The commercial vitality of the research unit in the street market was calculated by matching stores to the corresponding street unit and then performing the weighted sum of the number of stores in the street market unit, with V as the weight of the economic contribution of the stores.

#### 3.3.3. Independent variable.

The process of identifying indicators influencing vitality involved two main steps. First, studies related to the vitality of street markets were retrieved to establish a foundational understanding. Given the complex nature of street markets, as discussed in Section 2.2, the search query (“street market” OR “street” OR “commercial space” OR “public space”) AND “vitality” was applied to the Topic field in the Web of Science database. This resulted in the selection of 30 relevant papers for variable identification. Second, indicators and categories were identified. Street markets are conceptualized as organic systems that integrate macro-structural designed spaces with micro-experiential spaces formed by daily activities. Consequently, research categories were divided into urban morphology, urban functionality, and human perception. High-frequency indicators suitable for street market environments were initially identified through literature review and field investigation. Given the abundance of morphological and functional indicators, SNA was used to determine the most critical indicators by calculating objective weights through centrality analysis, specifically betweenness centrality. The betweenness centrality is calculated as follows:


$$C_{B}\left(n_{i}\right)=\sum_{j<k}\frac{g_{jk}\left(n_{i}\right)}{g_{jk}}$$ (3)

where, $C_{B}\left(n_{i}\right)$ refers to the betweenness centrality of node $n_{i}$. $g_{jk}$ represents the total number of connections between nodes $n_{j}$ and $n_{k}$. $g_{jk}\left(n_{i}\right)$ indicates the count of paths passing through node $n_{i}$ that connect $n_{j}$ and $n_{k}$. i, j, and k denote the observation nodes.

**Fig 5:**
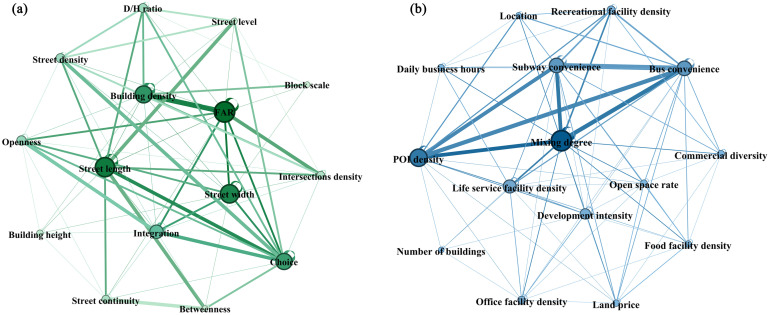
Network communities: (a) Urban morphology; (b) Urban functionality. Node size denotes betweenness centrality and edge thickness shows association strength. Key variables were selected based on centrality thresholds.

Gephi software was employed to calculate and normalize the betweenness centrality for each indicator to derive their objective weights. The visual network analysis results are presented in Fig 5. Six functional indicators with betweenness centrality values exceeding 2 and six morphological indicators with values exceeding 2.8, along with four human perception indicators identified earlier, were selected as the key independent variables. These 16 indicators are detailed in Table 1, representing the primary variables in our study.

**Table 1:** Independent variables and formulas.

Category	Indicator and formula	Explanation
Urban morphology	$FAR=\frac{\sum_{t=1}^{n}S_{t}}{S_{unit}}$	$S_{t}$ The total area of the buildings in the research unit $S_{unit}$The area of the research unit
	$Building\;density(\text{Bd})=\frac{\sum_{b=1}^{n}S_{b}}{S_{unit}}$	$S_{b}$ The area of the first floor
	$Street\;length(\text{Sl})=L_{r}$	$L_{r}$ The length of the center line of the research unit
	$Street\;width\;(\text{Sw})=\frac{S_{unit}}{L_{r}}$	
	$\text{Integration}\;(\text{Int})=\frac{\left(n-1\right)}{\sum_{i=1}^{n-1}D(p,i)}$	D(p,i) The shortest distance between nodes p and i n The total number of nodes in the network
	$\text{Choice}\;(\text{Cho})=\sum_{i\neq j}\frac{\sigma_{ij}(p)}{\sigma_{ij}}$	p a path or a node $\sigma_{ij}$ The total number of all shortest paths from node i to node j $\sigma_{ij}(p)$The number of shortest paths from node i to node j that pass through p
Urban functionality	$POI\;density(\text{POI})=\frac{N}{S_{unit}}$	N The number of POI facilities within the research unit
	$Mixing\;degree\;(\text{Mix})=-\sum_{i=1}^{n}\frac{Pi*\text{ln}\left(Pi\right)}{\text{ln}(n)}$	Pi The proportion of the i-th POI within the research unit n The number of POI types within the research unit.
	$\text{Life}\;\text{service}\;\text{facility}\;\text{density}\;(\text{Lsfd})=\frac{N_{l}}{S_{unit}}$	$N_{l}$ The number of life service facilities within the research unit
	$\text{Subway}\;\text{convenience}\;(\text{Sub})=L_{sta}$	$L_{sta}$ The shortest path distance from the midpoint of the research unit to the nearest subway station.
	$\text{Bus}\;\text{convenience}\;(\text{Bus})=\frac{N_{bus}}{S_{unit}}$	$N_{bus}$ The number of bus stations within 300 m around the research unit
	$\text{Development}\;\text{intensity}\;(\text{Dev})=\frac{\sum_{t=1}^{n}S_{t}}{\sum_{b=1}^{n}S_{b}}$	
Human perception	$Green\;vision\;rate(\text{Gre})=\frac{N_{gre}}{N_{tot}}*100\%$	$N_{gre}$ The number of greenery pixels in street view images $N_{tot}$ The number of total pixels in street view images
	$Enclosure\;degree(\text{En})=\left(1-\frac{N_{sky}}{N_{tot}}\right)*100\%$	$N_{sky}$ The number of sky pixels in street view images
	$Walkability\;(\text{Wal})=\frac{N_{sw}}{N_{tot}}*100\%$	$N_{sw}$ The number of sidewalk, floor and road pixels in street view images
	$Facility\;visibility(\text{Fac})=\frac{N_{fa}}{N_{tot}}*100\%$	$N_{fa}$ The number of signal lights, chair, and other street facilities pixels in street view images

**Fig 6:**
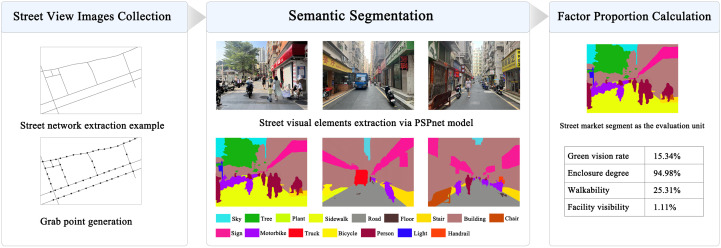
Workflow for street view image collection and analysis. Street view images are collected at sampled points, semantically segmented using PSPNet, and quantitatively analyzed for key visual environmental indicators.

Urban morphology indicators include FAR and building density, reflecting land use intensity and spatial efficiency [9], along with street length and width to indicate street network configuration [13]. Integration and choice measured in space syntax segment analysis [12,45], with radius set at 800 meters, are also included. To comprehensively assess urban functionality, key factors include POI density and mixing degree, which reflect amenity concentration and diversity [11]; subway and bus convenience, evaluating public transport accessibility [12]; development intensity, indicating economic activity and land use efficiency [9]; and life service facility density, assessing essential service availability. Human perception indicators encompass streetscape visual elements like green vision rate, enclosure, walkability, and facility visibility [11,46]. Fig 6 details the extraction process for streetscape features. In March 2024, high-resolution cameras were employed to collect streetscape images from Shangxia Sha urban village street markets, addressing limitations of platforms like Baidu and Gaode Maps. Sampling points were set at the midpoint of main street in research units, capturing four viewing directions (0°, 90°, 180°, 270°). A total of 2,032 images were collected. To improve segmentation accuracy, the PSPNet algorithm, using a pyramid pooling module for multiscale contextual information, was employed. The ADE20k dataset, with over 25,000 annotated images, was used for training, and the collected images were analyzed using the trained network. Finally, the proportions of green vision rate, enclosure degree, walkability, and facility visibility within the streetscape were calculated.

### 3.4. Regression models

#### 3.4.1. GTWR model.

To analyze social vitality, we applied the GTWR model. The GTWR model is an extension of the GWR model that incorporates both spatial and temporal dimensions. This model allows for the analysis of spatially non-stationary relationships over time, providing a more comprehensive understanding of the spatial-temporal dynamics in the data [47]. Social vitality is dynamic, influenced by both location and timing of social activities, making the GTWR model ideal for accurately mapping its changes over time and space. The GTWR model can be expressed as:


$$y_{i}=\beta_{\text{0}}(u_{i},{\;v}_{i},{\;t}_{i})+\sum_{k=\text{1}}^{p}\beta_{k}(u_{i},{\;v}_{i},{\;t}_{i})x_{ik}+\epsilon_{i}$$ (4)

Where $\left(u_{i},{\;v}_{i}\right)$ represents the spatial geographic location of the sample points and $t_{i}$ is the time at observation point i, $x_{ik}$ is the covariate, $\beta_{k}$ represents the k-th local regression coefficient, and $\epsilon_{i}$ is the model regression residual.

#### 3.4.2. MGWR model.

For assessing commercial vitality, MGWR model were employed. The MGWR model is an advanced form of GWR that allows for the assessment of spatial processes operating at different scales [48]. This adaptability helps identify specific spatial factors affecting commercial vitality at different scales, resulting in a more precise analysis. By enabling each explanatory variable to have its own bandwidth, MGWR captures the varying spatial scales at which different processes occur. The MGWR model can be expressed as:


$$y_{i}=\sum_{k=1}^{p}\beta_{b_{w}k}(u_{i},{\;v}_{i})x_{ik}+\epsilon_{i}$$ (5)

where $x_{ik}$ is the covariate, $\left(u_{i},{\;v}_{i}\right)$ represents the spatial geographic location of the sample points, $\beta_{b_{w}k}$ refers to the bandwidth of the regression coefficient for the k-th variable, and $\epsilon_{i}$ is the model regression residual.

## 4. Results

### 4.1. Descriptive analysis for street markets in the Shangxia Sha urban village

**Fig 7:**
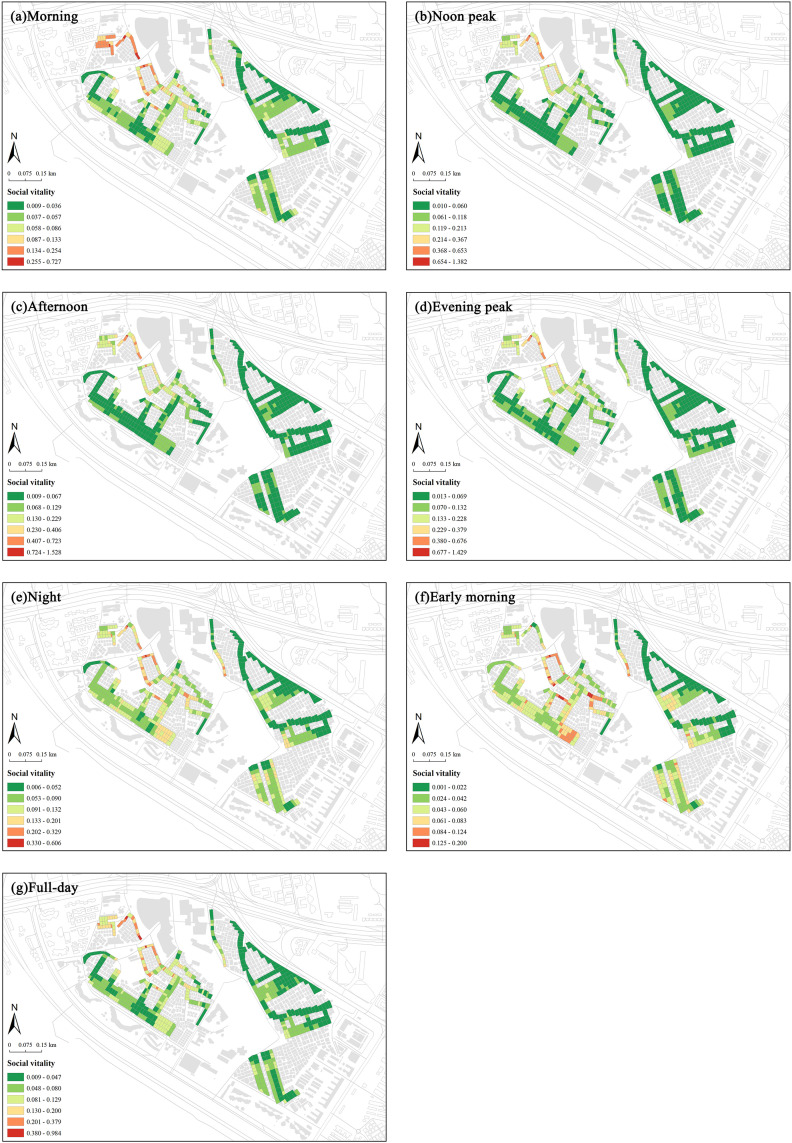
Spatiotemporal distribution of social vitality within the street markets of Shangxia Sha urban village. Maps illustrate the spatial distribution of social vitality within the street markets during seven time periods: (a) morning; (b) noon peak; (c) afternoon; (d) evening peak; (e) night; (f) early morning; (g) full-day average. The basemap, including roads and building outlines, is based on data from OpenStreetMap contributors (Open Database License, ODbL). Building outlines were further supplemented and corrected by the authors through field surveys. Colored research units were created by the authors in GIS software.

Social vitality analysis, based on 24-hour temporal characteristics of two weeks data, revealed minimal street activities between 3:00 and 6:00. Therefore, data from this period were excluded to avoid bias. We constructed six time-segment models based on 24-hour key nodes: daytime segments include morning (7:00–10:00), noon peak (11:00–13:00), and afternoon (14:00–16:00); nighttime segments include evening peak (17:00–20:00), night (21:00–23:00), and early morning (24:00–2:00). Additionally, a comprehensive analysis was conducted to encompass the entire day. Each segment’s social vitality is averaged from the hourly vitality within that segment. The natural breaks method was used to classify the values of social and commercial vitality, as well as built environment indicators, in the street markets of the Shangxia Sha urban village, revealing their spatial distribution characteristics. Significant spatial clustering is seen in social vitality across all periods (Fig 7). In the morning, noon peak, afternoon, and evening peak, the northwest region shows notably higher vitality. At night and early morning, the western region stands out. The northwest of Shangxia Sha urban village, with many dining facilities and convenience stores, along with two large shopping malls, boosts social vitality. The southwestern region’s variety of commercial establishments, like clothing stores, supermarkets, restaurants, and fruit shops, meets diverse needs, creating a commercial hub that attracts foot traffic and stimulates commercial activity. Comparing average social vitality across periods, the evening peak has the highest value, while early morning is lowest. The evening peak is marked by increased leisure and consumption activities after work, with residents dining, shopping, or socializing, especially in commercially rich areas. In early morning, most people rest, and minimal commercial and social activities result in low vitality.

**Fig 8:**
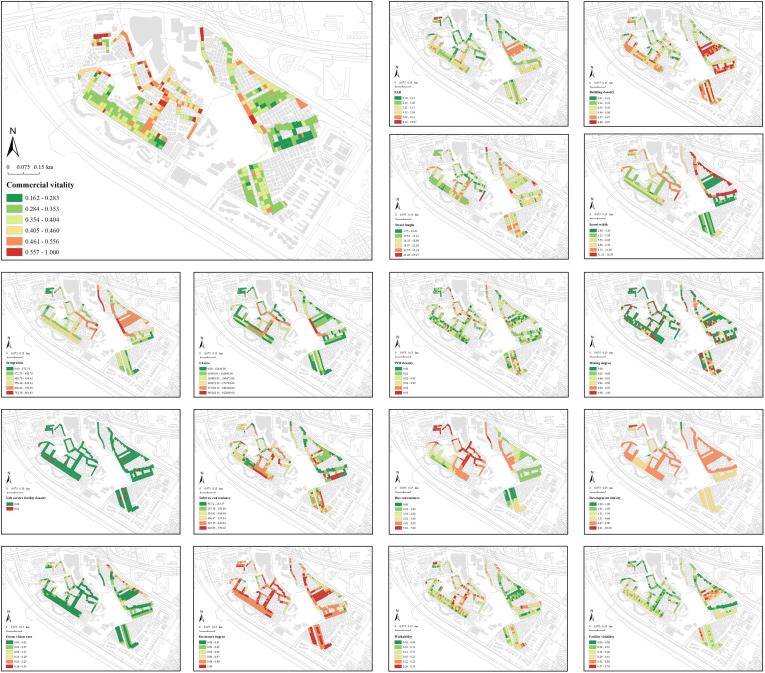
Spatial distribution of commercial vitality and built environment factors within the street markets of Shangxia sha urban village. The basemap, including roads and building outlines, is based on data from OpenStreetMap contributors (Open Database License, ODbL). Building outlines were further supplemented and corrected by the authors through field surveys. Colored research units were created by the authors in GIS software.

Fig 8 shows the spatial distribution of commercial vitality and selected built environment variables, revealing observable spatial differences. Commercial vitality is generally dispersed, with higher values in the western and northern regions due to denser large-scale shopping and dining facilities meeting residents’ daily needs.

### 4.2. Preliminary test for regression

Before regression analysis, each indicator was normalized. Subsequently, this study calculated Moran’s I statistics for the dependent variables, including commercial vitality and social vitality over different periods, to assess spatial autocorrelation. As shown in Table 2, Moran’s I statistics for both commercial and social vitality are significant at the 1% level across all periods, with values exceeding 0.66. These results indicate significant spatial clustering, with areas of similar vitality levels being geographically close.

**Table 2:** Results of global spatial autocorrelation analysis.

Category	Indicator	Moran’s *I*	Z	P
Social vitality	Morning	0.685	20.709	0.000
	Noon peak	0.707	21.934	0.000
	Afternoon	0.707	21.947	0.000
	Evening peak	0.695	21.391	0.000
	Night	0.668	19.641	0.000
	Early morning	0.698	20.378	0.000
Commercial vitality	Overall	0.535	15.654	0.000

Multicollinearity analyses were used to assess model reliability. Variables with a variance inflation factor (VIF) over 10 were excluded. Except for FAR and building density, 14 variables had VIF values under 10. Based on findings of related research, building density impacts urban vitality more than FAR [9], so FAR was removed. Further multicollinearity analysis showed all 15 remaining variables had VIF values below 10 (Table 3), and were used for MGWR and GTWR modeling.

**Table 3:** Results of variable collinearity diagnostics.

Category	Indicator	Tolerance	VIF
Urban morphology	Building density	0.522	1.917
	Street length	0.901	1.109
	Street width	0.373	2.680
	Integration	0.495	2.022
	Choice	0.412	2.429
Urban functionality	POI density	0.593	1.686
	Mixing degree	0.628	1.592
	Life service facility density	0.906	1.104
	Subway convenience	0.815	1.228
	Bus convenience	0.632	1.583
	Development intensity	0.889	1.125
Human perception	Green vision rate	0.560	1.786
	Enclosure degree	0.669	1.495
	Walkability	0.568	1.762
	Facility visibility	0.414	2.418

### 4.3. Regression analysis

#### 4.3.1. Spatiotemporal heterogeneity in the effects of built environment factors on social vitality via GTWR.

To validate the GTWR model’s applicability, OLS, GWR, and GTWR were applied to calculate social vitality and its influencing factors. Results show the GTWR model achieves the highest adjusted R² (0.755) and the lowest AICc, outperforming OLS and GWR (Table 4).

**Table 4:** Comparison of OLS, GWR, and GTWR fitting results.

Regression model	AICc	R²	Adjusted R²
OLS	7512.253	0.319	0.316
GWR	5569.744	0.696	0.669
GTWR	3630.626	0.756	0.755

The average coefficients of various factors across different time periods in the GTWR analysis are used to compare the impact of different indicators on social vitality. As shown in Table 5, Bus convenience exhibits the highest positive correlation coefficient, followed by green vision rate, POI density, subway convenience, and integration. Street length shows the highest negative correlation coefficient, followed by building density, development density, mixing degree, street width, facility visibility, choice, walkability, life service facility density, and enclosure degree.

**Table 5:** Descriptive statistics of average GTWR coefficients estimation across time periods.

Category	Indicator	Morning	Noon peak	Afternoon	Evening peak	Night	Early moring	Overall
Urban morphology	Building density	−0.128	−0.148	−0.160	−0.152	−0.099	−0.074	−0.127
	Street length	−0.364	−0.428	−0.468	−0.448	−0.316	−0.251	−0.379
	Street width	−0.065	−0.061	−0.066	−0.081	−0.091	−0.083	−0.074
	Integration	0.025	0.042	0.040	0.007	−0.044	−0.050	0.003
	Choice	−0.070	−0.101	−0.106	−0.068	0.009	0.025	−0.052
Urban functionality	POI density	0.109	0.139	0.155	0.147	0.101	0.079	0.122
	Mixing degree	−0.083	−0.099	−0.105	−0.096	−0.063	−0.049	−0.083
	Life service facility density	−0.023	−0.026	−0.029	−0.033	−0.033	−0.029	−0.029
	Subway convenience	0.027	0.044	0.047	0.031	−0.002	−0.009	0.023
	Bus convenience	0.191	0.223	0.232	0.203	0.123	0.092	0.177
	Development intensity	−0.049	−0.088	−0.111	−0.114	−0.076	−0.058	−0.083
Human perception	Green vision rate	0.130	0.168	0.184	0.165	0.090	0.060	0.133
	Enclosure degree	−0.010	−0.016	−0.015	−0.011	−0.001	0.002	−0.008
	Walkability	−0.044	−0.066	−0.073	−0.058	−0.019	−0.007	−0.044
	Facility visibility	−0.054	−0.088	−0.101	−0.084	−0.034	−0.020	−0.064

**Fig 9:**
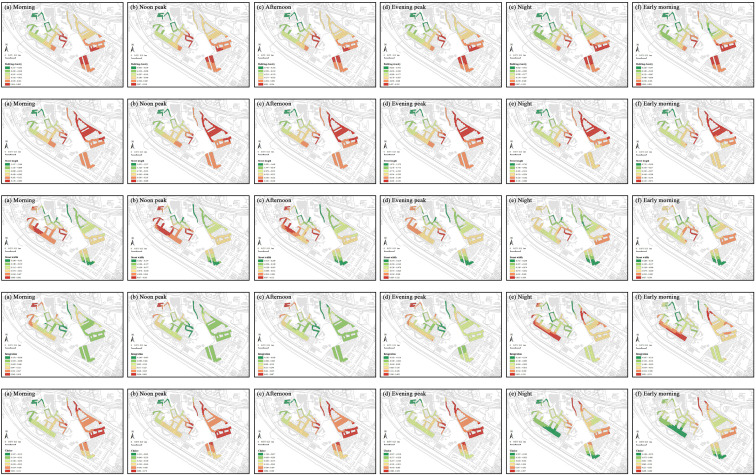
Spatial distribution of GTWR coefficients for urban morphology indicators. Each subfigure displays the GTWR coefficient values for key urban morphology indicators (building density, street length, street width, integration and choice) at six time periods: (a) morning, (b) noon peak, (c) afternoon, (d) evening peak, (e) night, and (f) early morning, revealing their spatial heterogeneity and temporal dynamics within the street markets. The basemap, including roads and building outlines, is based on data from OpenStreetMap contributors (Open Database License, ODbL). Building outlines were further supplemented and corrected by the authors through field surveys. Colored research units were created by the authors in GIS software.

**Fig 10:**
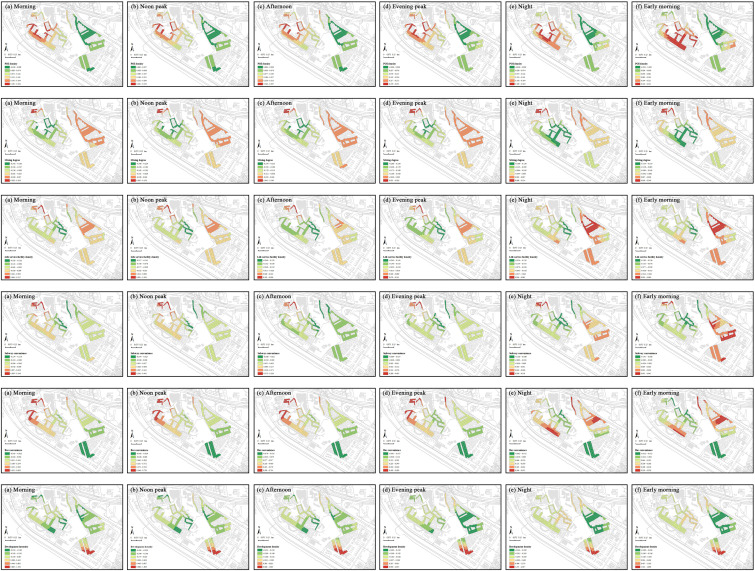
Spatial distribution of GTWR coefficients for urban functionality indicators. Each subfigure displays the GTWR coefficient values for key urban functionality indicators (POI density, mixing degree, life service facility density, subway convenience, bus convenience, and development intensity) at six time periods: (a) morning, (b) noon peak, (c) afternoon, (d) evening peak, (e) night, and (f) early morning, revealing their spatial heterogeneity and temporal dynamics within the street markets. The basemap, including roads and building outlines, is based on data from OpenStreetMap contributors (Open Database License, ODbL). Building outlines were further supplemented and corrected by the authors through field surveys. Colored research units were created by the authors in GIS software.

**Fig 11:**
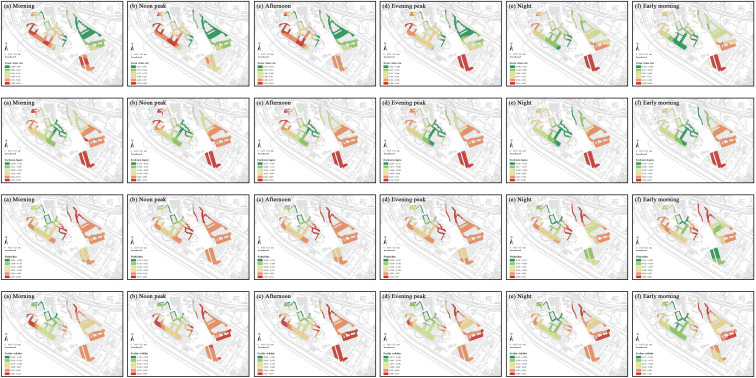
Spatial distribution of GTWR coefficients for human perception indicators. Each subfigure displays the GTWR coefficient values for key human perception indicators (green vision rate, enclosure degree, walkability, and facility visibility) at six time periods: (a) morning, (b) noon peak, (c) afternoon, (d) evening peak, (e) night, and (f) early morning, revealing their spatial heterogeneity and temporal dynamics within the street markets. The basemap, including roads and building outlines, is based on data from OpenStreetMap contributors (Open Database License, ODbL). Building outlines were further supplemented and corrected by the authors through field surveys. Colored research units were created by the authors in GIS software.

The average coefficient for each period indicates whether the influence of the indicator on social vitality is positive or negative during that period (Table 5). This study applied the natural breaks method to classify the coefficients of independent variables over different time periods in the GTWR model, highlighting the dynamic spatiotemporal variations in regression coefficients for various built environment factors, as shown in Figs 9–11.

The spatial distributions of building density and street length effects remain consistent throughout the day. High-coefficient areas for building density are in the central and southeast, and for street length in the east. Low-coefficient areas for building density are northwest, and for street length in the west. Both negatively impact social vitality, with the strongest effects in the afternoon and the weakest at early morning. High building density leads to overcrowding and reduces open spaces for social interactions, while overly long streets reduce accessibility and walkability, discouraging pedestrian activity and hindering efficient urban connectivity, particularly during peak times. Street width, integration, and choice exhibit temporal variations in their spatial effects. Street width’s high-coefficient areas are in the west during the daytime and shift to the central at the nighttime, while low-impact areas remain consistently in the north and southeast throughout the day. Street width negatively impacts social vitality, with the strongest negative effect observed at night due to safety concerns, and the weakest effect occurring during the noon peak, when better lighting and higher activity levels mitigate the impact. Integration’s high-coefficient zones are in the northwest from morning to evening peak, shifting to the southwest and northeast at night and early morning. Its low-coefficient zones are central from morning to evening peak, moving northwest at night and early morning. Integration’s effects polarize, being most positive during the noon peak with increased accessibility and negative at night due to low resource use. Choice’s high-coefficient areas are in the east from morning to evening peak, shifting northeast at night and early morning, while low-coefficient areas move from northwest from morning to evening peak to south at night and early morning. Choice has the strongest positive impact at night, offering flexibility, but complicates traffic during afternoon peaks.

The spatial distribution of POI density and mixing degree are stable throughout the day. High-coefficient areas for POI density are in the west, and for mixing degree in the northwest. Low-coefficient areas for POI density are in the east, and for mixing degree in the southwest. POI density positively impacts social vitality, peaking in the afternoon as commercial activities attract foot traffic but weakening early in the morning when activities cease. In contrast, the mixing degree’s impact is predominantly negative, strongest in the afternoon during peak traffic and reduced in the early morning due to lower activity levels. The spatial effects of life service facility density, subway convenience, bus convenience, and development intensity vary temporally. High-coefficient areas for life service facility density shift from the northwest from morning to evening peak to the northeast at night and early morning, while low-coefficient areas remain central. High-coefficient areas of development intensity remain in the southeast, whereas low-coefficient areas shift from the central and northwest during the day to the northeast at night. Life service facility density and development intensity primarily negatively affect vitality, peaking during the evening rush due to overcrowding and resource stress. Subway convenience has high-coefficient areas in the northwest from morning to night, moving to the east at early morning, while low-coefficient areas remain central. Subway convenience has a polarizing effect, peaking positively in the afternoon due to enhanced accessibility but turning negative early in the morning as services reduce. Bus convenience’s high-coefficient areas shift from the northwest from morning to evening peak to the southwest and northeast at night and early morning, with low-coefficient areas consistently in the southeast. Bus convenience positively impacts vitality, strongest in the afternoon and weakest in the early morning due to similar service patterns of subway. Development intensity consistently shows high-coefficient areas in the southeast, and low-coefficient areas in the center during the daytime and the east during the nighttime. High development in urban villages causes crowding, peaking in negative impact during evening traffic and easing with lighter morning flow.

Green vision rate and walkability show temporal variations in their spatial impacts. Green vision rate has high-coefficient areas in the west during the daytime, moving to the southeast during the nighttime, while low-coefficient areas in the northeast from morning to evening peak, moving to the southwest at night and early morning. Green vision rate positively impacts vitality, strongest in the afternoon due to sunlight and diverse outdoor activities and weakest in the early morning due to reduced activity and visibility. Walkability consistently shows high-impact areas in the central region, with low-impact areas in the northwest from morning to night, and in both the northwest and southeast at early morning. Walkability negatively impacts vitality, peaking in the afternoon with congestion and heat but weakening in the early morning with smoother traffic. The spatial distributions of enclosure degree and facility visibility effects remain consistent throughout the day. High-coefficient areas for enclosure degree are in the southeast, and for facility visibility in the southwest and east. Low-coefficient areas are central for enclosure degree and northwest for facility visibility. Enclosure degree’s effects polarize, being most positive early in the morning due to the sense of safety and community it provides and most negative at noon due to overcrowding and restricted movement. Facility visibility negatively impacts vitality, strongest in the afternoon due to congestion and pressure from dense facility use, but weakens in the early morning as utilization decreases.

#### 4.3.2. Spatial heterogeneity and multiscale effects of built environment factors on commercial vitality via MGWR.

Table 6 presents indicators for commercial vitality using OLS, GWR, and MGWR models. The MGWR model shows a smaller AICc, higher adjusted R², and a wider range of variable bandwidths, indicating a better fit compared to the other models. Variable bandwidth reflects the spatial scale of impact for various factors. Larger scales suggest less spatial heterogeneity, while smaller scales indicate more. For commercial vitality, the spatial scales for factors like street width, integration, and enclosure degree are 92, 68, and 44, respectively, showing high spatial heterogeneity. Conversely, factors like building density, mixing degree, and bus convenience have scales of 506, nearly matching the sample size, indicating they are global variables with uniform effects across the study area.

**Table 6:** Comparison of OLS, GWR, and MGWR fitting results.

Regression model	Rss	AICc	R²	Adjusted R²	Bandwidth	
OLS	286.566	1186.055	0.436	0.419	–	
GWR	148.440	1078.498	0.708	0.633	152	
MGWR	141.302	1005.607	0.722	0.664	Building density	506
					Street length	286
					Street width	92
					Integration	68
					Choice	506
					POI density	128
					Mixing degree	506
					Life service facility density	284
					Subway convenience	266
					Bus convenience	506
					Development intensity	234
					Green vision rate	140
					Enclosure degree	44
					Walkability	268
					Facility visibility	450

Table 7 presents the statistical descriptions of coefficients from the MGWR analysis. At the 95% confidence interval, factors like building density, choice, mixing degree, subway convenience, bus convenience, and facility visibility are insignificant for commercial vitality, while other variables show localized significance. This lack of statistical significance may be attributable to spatial heterogeneity and local contextual differences, where the effects of these variables vary across locations and are diluted when averaged across the study area. For example, transportation convenience variables such as subway and bus accessibility might have a significant impact on commercial vitality only in specific neighborhoods with distinct transit patterns. However, when analyzed at the broader scale, these localized effects may not be strong enough to reach statistical significance across the entire study area. The average coefficients are used to reveal and compare the impact of various factors on commercial vitality. POI density exhibits the highest positive correlation with commercial vitality, followed by integration, green vision rate, street length, and walkability. Conversely, life service facility density has the strongest negative correlation, followed by street width, development intensity, and enclosure degree.

**Table 7:** Statistical analysis of regression results in the MGWR model.

Category	Indicator	Mean	STD	Min	Median	Max	p ≤ 5(%)
	Intercept	−0.240	0.433	−0.874	−0.221	0.457	77.165%
Urban morphology	Building density	0.038	0.005	0.033	0.035	0.046	0.000%
	Street length	0.092	0.043	−0.016	0.111	0.140	68.307%
	Street width	−0.036	0.176	−0.457	0.003	0.496	7.283%
	Integration	0.154	0.257	−0.588	0.173	0.600	28.937%
	Choice	0.026	0.008	0.014	0.026	0.038	0.000%
Urban functionality	POI density	0.257	0.129	0.123	0.225	0.704	95.276%
	Mixing degree	0.032	0.019	0.006	0.028	0.055	0.000%
	Life service facility density	−0.095	0.044	−0.136	−0.108	0.035	61.811%
	Subway convenience	0.014	0.033	−0.051	0.012	0.066	0.000%
	Bus convenience	0.008	0.007	−0.002	0.011	0.018	0.000%
	Development intensity	−0.035	0.183	−0.418	−0.018	0.251	35.433%
Human perception	Green vision rate	0.097	0.225	−0.258	0.066	0.542	37.008%
	Enclosure degree	−0.005	0.344	−0.721	−0.036	1.198	27.953%
	Walkability	0.030	0.045	−0.073	0.025	0.112	1.772%
	Facility visibility	0.017	0.043	−0.032	0.018	0.077	0.000%

**Fig 12:**
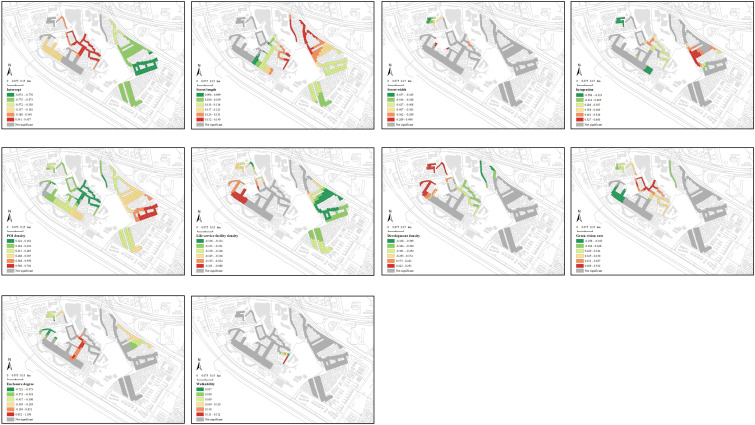
Spatial patterns of coefficients in the MGWR model. Each panel shows the spatial patterns of MGWR coefficients for the following variables: intercept, street length, street width, integration, POI density, life service facility density, development density, enclosure degree, walkability, and green vision rate. The basemap, including roads and building outlines, is based on data from OpenStreetMap contributors (Open Database License, ODbL). Building outlines were further supplemented and corrected by the authors through field surveys. Colored research units were created by the authors in GIS software.

Using the natural breaks method, this study categorized the coefficients of independent variables derived from the MGWR model, emphasizing the distinct spatial heterogeneity in regression coefficients for various built environment factors (Fig 12). Within the morphological features, street length emerges as a positive determinant of commercial vitality, with coefficient values ranging from 0.090 to 0.140, as longer streets in dense urban villages disperse pedestrian flow and support diverse commercial activities. Street width has mixed effects, with coefficient values ranging from −0.457 to 0.456. Wider streets benefit high-traffic businesses like dining and retail, while narrower streets suit community-oriented small shops by fostering customer interaction. Integration also varies widely, with coefficient values ranging from −0.588 to 0.600. In some areas, high integration improves accessibility and commerce, while in others, it causes traffic and environmental issues, hindering vitality.

POI density, a critical functional indicator, has a positive correlation, with coefficients ranging between 0.123 and 0.704, as concentrated commercial facilities attract consumers. Meanwhile, life service facility density shows a negative correlation, with coefficients ranging between −0.136 and −0.088, as it competes for limited space, reducing commercial diversity. Development density has mixed effects, with coefficients ranging between −0.418 and 0.251. While higher density can boost consumer demand, it may also lead to congestion and resource competition.

Human perception indicators demonstrate spatial heterogeneity. Green vision rate has mixed effects, with coefficients ranging between −0.258 and 0.542. Greenery improves environmental quality, attracting consumers, but excessive greenery can limit commercial space. Enclosure degree also varies widely, with coefficients ranging between −0.721 and 1.198. This creates a favorable atmosphere for small businesses but excessive confinement reduces consumer activity. Walkability consistently supports commercial vitality, with coefficients ranging between 0.107 and 0.112, by encouraging frequent visits.

## 5. Discussion

This study adopts a meso- and micro-scale approach, focusing on Shangxia Sha, a typical urban village street market, to explore complex socio-economic and spatial interactions often overlooked in broader urban analyses. It examines both the social vitality and the economic vitality of the market, as well as their respective relationships with the built environment, by applying the GTWR and MGWR models. These methods capture spatiotemporal heterogeneity and multi-scale spatial heterogeneity, which were not addressed in previous studies on homogeneous street environments. To address gaps in existing research on the built environment, the study integrates macro-structural and micro-experiential analyses, using street view imagery as a key tool for micro-experiential insights. Given the lack of street view data for urban village streets on major open-source platforms, pedestrian-collected images were used. Compared to traditional vehicle-based methods, this approach offers a more accurate and immersive representation of the market environment. This enhances data accuracy and supports a more precise evaluation of the multidimensional relationships between the built environment and multidimensional vitality.

### 5.1. Comparative analysis of determinants of social and commercial vitality of the street markets

Results reveal three key aspects of the built environment are linked to varying levels of social and commercial vitality. Specifically, functional indicators play a pivotal role in enhancing the social and commercial vitality of street markets, exerting greater influence than other indicators. This aligns with prior studies emphasizing the importance of functional attributes in fostering social vitality [9], while highlighting their unique contribution to commercial vitality. Accessibility to public transportation, such as buses and subways, emerges as a key driver, significantly boosting both forms of vitality. Empirical evidence supports this, showing that dining, retail, and leisure facilities cluster in areas with convenient transportation, attracting higher foot traffic and economic activity [29,49]. This can also be explained from a spatial behavior perspective: improved public transport accessibility reduces travel costs and time, thereby promoting frequent and diverse interactions among residents and visitors, which in turn supports both social exchange and commercial transactions. Functional density also promotes social and commercial vitality by concentrating resources and opportunities, enhancing residents’ quality of life and economic prospects, as demonstrated by the empirical research [33]. However, excessive functional mixing—common in high-density areas such as urban villages—can lead to environmental congestion and infrastructure overload, diminishing community cohesion and quality of life. The underlying causal mechanism may be that an overabundance of diverse functions intensifies competition for limited space and resources, leading to conflicts between different user groups and a decline in perceived neighborhood safety and comfort. This finding partly challenges Jane Jacobs’ positive view of mixed-use development [16], echoing recent concerns about its potential downsides [50]. Additionally, our findings support the conclusions of Mouratidis [34], demonstrating that excessive development intensity further undermines social and commercial vitality by exacerbating resource depletion, traffic congestion, and environmental degradation. This suggests that urban form and density must be carefully balanced to maximize benefits while minimizing negative externalities.

Morphological factors affecting social vitality largely align with existing literature while offering new insights into commercial vitality. Spatial integration from space syntax analysis significantly enhances vitality, as demonstrated in studies from cities like Wuhan, where integrated street networks promote urban development [12]. From an urban sociology perspective, streets with higher spatial integration tend to attract greater pedestrian flow and diverse activities. This increased movement and visibility create more opportunities for spontaneous social interactions, which can indirectly support the formation of social networks and a sense of community. Conversely, the Choice metric negatively impacts social vitality, consistent with prior findings [11]. At the street market scale, street length and width show contrasting effects: smaller blocks and more intersections stimulate neighborhood interaction, aligning with traditional urban design theories [16]. From the perspective of urban sociology and spatial behavior theory, such spatial configurations foster frequent, spontaneous encounters among residents, which in turn strengthen social ties and foster a sense of community. In contrast, while longer streets enhance commercial vitality by accommodating more shops and meeting diverse consumption needs in high-density environments, excessively long streets may reduce the likelihood of repeated interactions among residents. This spatial separation can weaken neighborhood cohesion, as greater distances discourage social engagement and diminish the informal surveillance and mutual support that underpin vibrant communities. Opposing the conclusions of related studies, building density negatively affects social vitality by restricting open spaces and reducing opportunities for interaction [9]. This can be attributed to the reduction of shared public spaces, which are crucial for spontaneous social behaviors and informal gatherings.

On the human perception dimension, this study expands existing dimensions by exploring its relationship with commercial vitality. The green vision rate significantly enhances both social and commercial vitality. While some studies suggest greenery may reduce social vitality [9], in the dense urban villages studied, greenery beautifies streetscapes, promotes well-being, attracts foot traffic, and extends shoppers’ stays, boosting their spending. Pedestrian-friendly street designs further enhance commercial vitality by improving accessibility to shops, though they may inadvertently reduce spaces for social interaction, weakening social vitality. This finding contradicts some studies [11] but aligns with others that highlight similar concerns [9]. The diversity of these research findings may stem from differences in research context, methodology, time span considerations, and other factors. Enclosure degree negatively impact both forms of vitality, heightening spatial oppression and limiting commercial diversity. This finding aligns with some empirical studies [11] and sheds light on its commercial implications. Additionally, the negative impact of facility visibility on social vitality highlights the trade-offs in high-density urban environments, where overly prominent facilities may occupy spaces better suited for diverse social or public uses.

### 5.2. Strategic implications for future planning and policy development

To foster vibrant, high-density urban public and commercial spaces, such as street markets, it is crucial to adopt a strategic framework that harmonizes various elements such as accessibility, spatial configuration, and experiential quality. By skillfully managing these aspects, urban planners can create lively and inclusive environments, particularly benefiting low-income communities and informal settlements. The complexity of urban systems requires mitigating risks such as excessive functional mixing and overdevelopment. Rational planning must focus on functional integration to ensure convenience while minimizing conflicts. Moderate development and facility enhancement can increase an area’s carrying capacity, but it is crucial to avoid surpassing reasonable thresholds which could lead to adverse outcomes. In high-density contexts, optimizing land use policies and infrastructure efficiency is paramount for fostering sustainable growth.

To translate these principles into effective practice, several actionable recommendations can be implemented: First, urban design should prioritize multimodal public transit access and maintain a balanced mix of land uses, avoiding excessive functional overlap. Second, policymakers are advised to regulate building densities to ensure the availability of open and flexible public spaces, which are vital for encouraging social interaction. Third, streetscape improvements—such as increasing greenery, enhancing walkability, and providing adequate street furniture—not only beautify the environment but also attract more visitors and support both social and commercial activities. Fourth, street governance measures, including flexible vendor management and clear spatial delineation for different uses, can reduce conflicts and enhance the overall functioning of street markets. Fifth, land use regulations should promote the adaptive reuse of existing structures, while ensuring that infrastructure provisions keep pace with development intensity to prevent overcrowding and resource depletion.

These actionable strategies are particularly relevant for low-income communities and informal settlements, where the vitality of street markets plays a pivotal role in enhancing community well-being and reducing social inequalities. By integrating these measures, urban planners can foster environments that achieve synergy between community interaction, economic growth, and environmental sustainability, ultimately contributing to a more equitable and resilient urban fabric.

## 6. Conclusion and limitations

This study examines the street markets in Shangxia Sha urban village of Shenzhen, a representative case of high-density and mixed used street markets undergoing rapid transformation. It investigates the spatiotemporal patterns and key influencing factors of the social and commercial vitality of street markets in urban villages. Using GTWR and MGWR models, the study captures both the spatiotemporal heterogeneity and the multi-scale spatial heterogeneity in the relationship between multidimensional vibrancy and the built environment. By doing so, this research addresses the gap in existing studies, which typically focus on analyzing homogeneous urban streets with global regression models and tend to emphasize isolated aspects of social vitality and the macro-structural built environment.

Integrating Baidu Huiyan population heat map data and Weibo heck-in data, we developed a comprehensive indicator to serve as a proxy for the social vitality of street markets. Detailed quantification of commercial vitality was achieved through the classification and strategic application of data from Dianping. By integrating macro-structural analysis with micro-experiential perspectives, a comprehensive framework of built environment for understanding street market vitality is established. At the macro level, we identified key morphological and functional indicators characterized by high betweenness centrality. At the micro level, human perception indicators were identified using insights from existing literature and street view imagery enhanced by deep learning. Through the application of GTWR to analyze social vitality and MGWR to assess commercial vitality, we gained deeper insights into the spatial differentiation and drivers and inhibitors of social and commercial vitality in street markets. The GTWR analysis results reveal that bus convenience exhibits the strongest positive correlation with social vitality, followed by green vision rate, POI density, subway convenience, and integration. Street length has the highest negative correlation coefficient, followed by building density, development density, mixing degree, street width, facility visibility, choice, walkability, life service facility density, and enclosure degree. The MGWR analysis results indicate that POI density exerts the strongest positive influence on commercial vitality, followed by integration, green vision rate, street length, and walkability. Conversely, life service facility density has the strongest negative impact, followed by development intensity and enclosure degree.

These comprehensive analytical results provide valuable insights into the multidimensional vitality relationships characterizing urban village street markets, offering a foundation for broader urban renewal strategies. Building on these findings, achieving sustainable urban renewal requires a balanced and integrative approach that harmonizes urban morphology, functionality and human perception. By addressing the intricate interplay of these factors, while mitigating risks such as excessive functional mixing and overdevelopment, urban planning can foster environments that are vibrant, inclusive, and adaptable to future challenges.

Three main limitations of this study should be noted. First, while Baidu Heat Maps and Weibo check-in data partially represent social vitality, they lack fine-grained details and do not account for user groups without smartphones, like the elderly and children. Dianping data mainly reflects fixed commercial activities, neglecting temporary ones like street vendors and night markets. Future research should integrate diverse data sources, including LBS and field data, to better capture both social and commercial vitality. In addition, a complementary qualitative comparative analysis approach could be employed in future studies to explore how different combinations of built environment factors jointly influence street market vitality. Second, while the focused analysis of Shangxia Sha offers unique insights into street markets, future studies could include multiple street markets to validate findings and enhance their applicability to diverse urban contexts. Third, the data collection was confined to a two-week period in March 2024. This interval was purposefully selected to avoid public holidays and to represent ordinary urban activity. Future research should extend data collection to cover multiple periods or seasons, which would help to assess and enhance the generalizability and robustness of the findings.

## Supporting information

S1 Data Datasets for street market vitality analysis. (XLSX)
